# Technologies Employed in the Treatment of Water Contaminated with Glyphosate: A Review

**DOI:** 10.3390/molecules25235550

**Published:** 2020-11-26

**Authors:** Patricio J. Espinoza-Montero, Carolina Vega-Verduga, Paulina Alulema-Pullupaxi, Lenys Fernández, Jose L. Paz

**Affiliations:** 1 Escuela de Ciencias Químicas, Pontificia Universidad Católica del Ecuador, Quito 17-01-2184, Ecuador; cbvegav@gmail.com (C.V.-V.); pauli95.alp@gmail.com (P.A.-P.); lmfernandez@puce.edu.ec (L.F.); 2 Departamento de Física, Escuela Politécnica Nacional, Ladrón de Guevara, Quito 17-12-866, Ecuador; jose.pazr@epn.edu.ec

**Keywords:** glyphosate, herbicides, commercial formulation, water pollution, water treatment process

## Abstract

Glyphosate [*N*-(phosphonomethyl)-glycine] is a herbicide with several commercial formulations that are used generally in agriculture for the control of various weeds. It is the most used pesticide in the world and comprises multiple constituents (coadjutants, salts, and others) that help to effectively reach the action’s mechanism in plants. Due to its extensive and inadequate use, this herbicide has been frequently detected in water, principally in surface and groundwater nearest to agricultural areas. Its presence in the aquatic environment poses chronic and remote hazards to human health and the environment. Therefore, it becomes necessary to develop treatment processes to remediate aquatic environments polluted with glyphosate, its metabolites, and/or coadjutants. This review is focused on conventional and non-conventional water treatment processes developed for water polluted with glyphosate herbicide; it describes the fundamental mechanism of water treatment processes and their applications are summarized. It addressed biological processes (bacterial and fungi degradation), physicochemical processes (adsorption, membrane filtration), advanced oxidation processes—AOPs (photocatalysis, electrochemical oxidation, photo-electrocatalysis, among others) and combined water treatment processes. Finally, the main operating parameters and the effectiveness of treatment processes are analyzed, ending with an analysis of the challenges in this field of research.

## 1. Introduction

In recent years, the global food demand caused by population growth has motivated an intensive use of agrochemicals in agricultural activities. However, this can cause an environmental impact [1,2]. One of the major worldwide concerns in our society is water pollution caused by pesticides, because of the potential adverse effects of these compounds on ecosystems and human health [3]. A type of pesticides frequently used in the agricultural areas are herbicides, which are used to kill or inhibit the normal growth of undesirable plants (also called pest) in crops [1,4]. Globally, glyphosate-based herbicides are the agrochemical most widely used because of their effectiveness in crops. This commercial herbicide was first marketed by Monsanto as Roundup in 1974 in the United States. Nowadays, it is commercialized by different names depending on the place [5,6]. In Ecuador, it is mostly commercialized by the name of Glifopac.

Glyphosate or [*N*-(phosphonomethyl)-glycine] has a highly effective foliar action herbicide because of its characteristics: I) It is a systemic herbicide because inhibits the 5-enolpiruvil-shikimato-3-phosphate-synthetase enzyme (EPSP), which is indispensable in essential amino acids synthesis for plant growth and development. This herbicide causes leaf decomposition and therefore the plant death between 5 to 30 days after application [6,7,8]; II) Post-emergent herbicide because it is applied to weeds in their first stages of development; III) Non-selective herbicide because it exerts action on all plant material with which it comes into contact [6,7,8]. This herbicide is commonly used for soil preparation before sowing, control of undesirable plants in crop areas, control of annual perennial special or invasive vegetation during post-harvest activities. In addition, it is used to control aquatic algae although the use of glyphosate herbicides is not approved for applications in the aquatic environment [4,9]. Because of its high solubility (12 g·L^−1^ at 25 °C) this herbicide can be easily detected in surface water and groundwater [4]. The surface water and groundwater contamination with glyphosate can occur through multiple routes: surface runoff, direct overspray, drift during herbicide application, improper application practices and disposal of herbicide wastes such as those originating from empty pesticide containers or fumigation equipment [6,10]. In this way, significant quantities of glyphosate, its coadjutants, and its degradation metabolites (such as Aminomethylphosphonic acid or AMPA, sarcosine, glycine, among others) reach aquatic environments; where due to its relatively long half-life (t_1/2_ glyphosate from 7 to 315 days, most commonly 45–60 days and t_1/2_ AMPA from 76 to 240 days), it can persistence in water [11,12]. For this reason, there are studies that demonstrate the presence of glyphosate in surface and groundwater [6,10].

Regarding ecotoxicology, once the herbicide (dose) enters the organism, it can have a toxic effect, which can be in the short (acute), medium (sub-lethal) and long term (chronic). Eco-toxicological studies have determined that glyphosate affects several aquatic trophic levels (freshwater/marine environments), being more toxic than the commercial formulations [12,13]. In terms of the risks posed to humans, the International Agency for Research on Cancer (IARC) of the World Health Organization (WHO) has placed the glyphosate herbicide into the 2A Group (probably carcinogenic to humans) and the Food and Agriculture Organization (FAO) reported that glyphosate and its major metabolite, aminomethylphosphonic acid (AMPA), are of potential toxicological concern, mainly as a result of the accumulation of residues in the food chain [14,15]. Based on health and environmental risks, it becomes necessary to develop strategies regarding water polluted with glyphosate, coadjutants, and metabolites. The conventional and non-conventional processes have been proposed as an alternative to treat water polluted with glyphosate [4]. However, physicochemical processes like coagulation, adsorption, and reverse osmosis, among others, are non-destructive and post-treatments of the adsorbent materials or solid wastes are necessary and costly [15,16]. On the other hand, biological processes can generate metabolites (e.g., AMPA) with higher toxicity potential if the operational conditions are not controlled [7]. In recent years, advanced oxidation processes (AOPs) have been proposed as an alternative treatment for water polluted with glyphosate [4]. The advantages of these technologies are related to the short residence times (minutes), reaching removal efficiencies higher than 90%, and a better ability to remove recalcitrant compounds (total mineralization).

This review describes the fundamental mechanism and presents some applications of these treatment processes studied for water polluted with glyphosate herbicide. It includes the basic concepts of each technique, operational conditions, and their effectiveness in water treatment. Conventional and non-conventional treatment processes are described. Biological treatment processes such as bacterial and fungi degradation, physicochemical processes—principally adsorption and membrane filtration—AOPs such as Fenton-based processes, UV-based process, and electrochemical oxidation like anodic oxidation and photoelectrocatalysis, and combined water treatment processes are analyzed. Finally, the challenges in this field of research are analyzed.

## 2. Water Treatment Processes to Remove Glyphosate from Water

### 2.1. Biologic Treatment Processes

Glyphosate biodegradation by microorganisms has been described as a safe, cost-effective and reliable means to remove this pollutant from soil and water [17]. Biodegradation occurs by bacterial or fungal species which generally use glyphosate as a source of nitrogen, carbon and phosphorous; after that, glyphosate is transformed into new compounds through different degradation pathways [18,19]. Even though glyphosate biodegradation depends on each microorganism metabolism, the degradation pathway involves enzymes able to cleavage of carboxymethylene–nitrogen (C–N) bond or carbon–phosphorus (C–P) bond in the molecule [7,20]. As a result, AMPA, sarcosine, and acetylglyphosate are formed as intermediate metabolites of degradation [7,18,19,20]. Different studies have reported effective and potential microorganisms for efficient and rapid bioremediation of glyphosate polluted environments. The work reported by Zhan et al. [7] summarizes some glyphosate-degrading strains (fungi and bacteria). Among glyphosate-degrading bacteria and glyphosate-degrading fungi are *Achromobacter* spp., *Agrobacterium radiobacter*, *Alcaligenes* sp. GL, *Arthrobacter* spp., *Bacillus cereus* CB4, *Ochrobactrum* spp., *Pseudomonas* spp., *Aspergillus niger*, *Aspergillus oryzae* A-F02, *Penicillium chrysogenum*, *Trichoderma harzianum*, among others.

**Figure 1:**
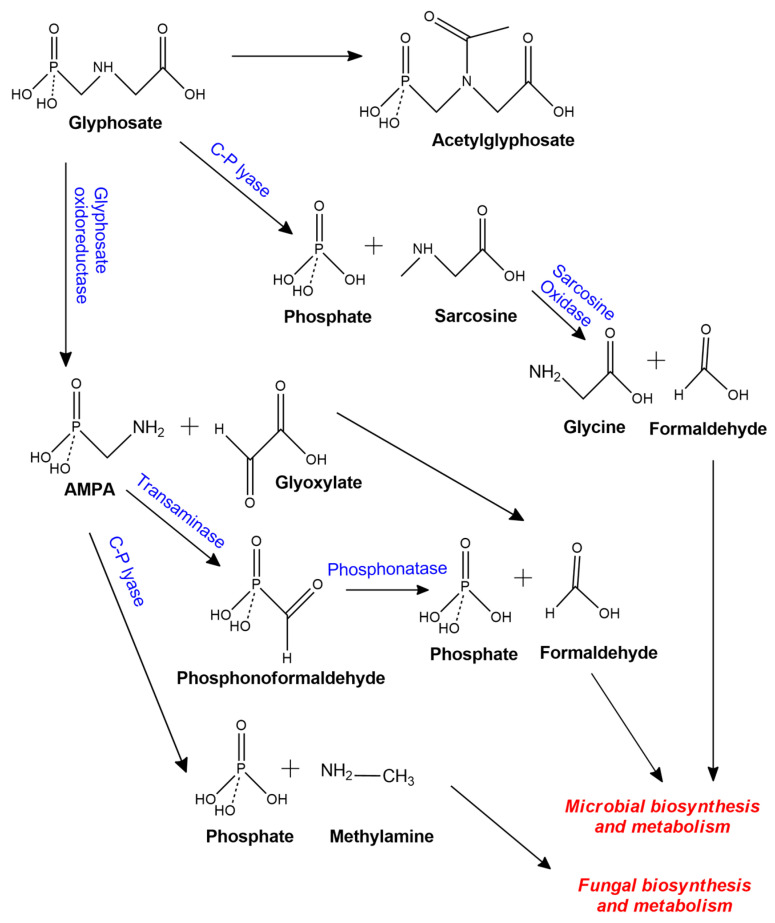
Degradation pathways of glyphosate in fungal and bacterial species [7,20,21].

Glyphosate biodegradation in bacterial species has three transformation pathways (Figure 1). The first pathway is the formation of AMPA and glyoxylate by the action of an enzyme called glyphosate oxidoreductase which cleavage C–N bond in the glyphosate molecule [7,17]. AMPA mostly serves as a substrate of enzymes that catalyze the cleavage of C–P bond producing methylamine and phosphate, or it can be metabolized to phosphonoformaldehyde by transaminase and then catabolized to formaldehyde and phosphate through phosphonatase enzyme. The glyoxylate molecule can be transformed into glycine and formaldehyde, and then used in microbial metabolism [17]. The second pathway is the formation of sarcosine and phosphate by the action of C–P lyase; after that, sarcosine oxidase transforms sarcosine to glycine and formaldehyde which are used for protein biosynthesis and metabolism in bacteria [7]. The third pathway converts glyphosate to acetylglyphosate and it cannot further utilize it as a nutrient source [7]. Among glyphosate-degrading bacteria are *Pseudomonas* sp., *Bacillus cereus*, *Arthrobacter* sp., *Ochrobactrum* sp., *Achromobacter* sp., *Alcaligensis* sp., *Flavobacterium* sp., *Agrobacterium radiobacter* sp., and others. Similar to bacteria, fungi species also utilize glyphosate as a source of phosphorus and nitrogen nutrients for their growth, and use analogous enzymes in the degradation pathway [21].

On the contrary, the fungi biodegradation pathway of glyphosate is shorter than the microbial pathway because fewer secondary metabolites are formed [21]. AMPA is the main metabolite, it is transformed to methylamine and then oxidized to formaldehyde through the action of methylamine dehydrogenase; finally, formaldehyde can be assimilated via the ribulose monophosphate cycle [20]. Only a few glyphosate-degrading fungi species have been reported, for example, *Trichoderma viridae*, *Aspergillus niger*, *Aspergillus Oryzae*, *Fusarium oxysporum*, *Trichoderma harzianum*, *Scopulariopsis* sp, *Penicillium citrinum.* Degradation pathways of glyphosate in fungi and bacterial species are summarized in Figure 1.

Based on the above, several authors have studied biological treatment to remove glyphosate and AMPA from water: *Flavobacterium* sp. was the first microorganism identified which can metabolize glyphosate, AMPA and sarcosine; it was applied in activated sludge to treat glyphosate-containing wastes at Monsanto Co. wastewater treatment plant [22]. *Agrobacterium radiobacter* was isolated from a bench-scale sequencing batch reactor degrading a waste stream containing glyphosate, it utilizes glyphosate as a sole source of carbon and energy [23]. *Pseudomonas* spp. is able to remove higher than 98% using a biofilm colonized with bacteria in immobilizing bacterial technology columns (IBT) [24]; a biological bed system using a bagasse-based bio-mix reaches 99% degradation of this herbicide after 6 months of treatment by bacterial action [25]; native bacteria from seawater are able to remove 48% of the initial concentration of glyphosate tested in batch culture [26]. *Pseudomonas* sp. and *Bacillus* sp. together are an effective microbial culture for biodegrading glyphosate in contaminated soils and aquifers [19]; unacclimated activated sludge culture tested in a batch culture showed its potential to biodegrade glyphosate under aerobic conditions and can be a good candidate for treating waters contaminated with high levels of glyphosate [27].

In the most recent study, Singh et al. [28] evaluated three isolated bacterial strains (*Streptomyces* sp., *Bacillus subtilis*, *Rhizobium leguminosarum*), which were useful in bioremediation of glyphosate polluted environment [28]. Fungal species also have shown glyphosate degradation activity in aqueous media: *Fusarium oxysporum* strains were capable of biodegrading glyphosate in pure and consortium cultures in a platform shaker and batch bioreactor, they achieved about 41% degradation efficiency after 5 days of culture [29]. *Trichoderma harzianum* has been found to have AMPA-degrading activity and achieved 69% of AMPA degradation after 10 days of incubation [30], a recent study reports the efficiency of *Aspergillus oryzae* A-F02 isolated from an aeration tank in a pesticide factory, which can transform less than 50% of glyphosate to AMPA after 72 h of incubation [20].

As shown above, the biologic treatment appears to be a promising process to remove glyphosate from water. However, this process could present some disadvantages. First, to evaluate the potential of glyphosate-degrading microorganism, it is important to enhance some experimental conditions such as initial pH, incubation temperature, glyphosate concentration, inoculation biomass and incubation time, because all of them are important operational parameters to optimize bioremediation of real water polluted with glyphosate. Second, under controlled conditions, some microorganisms use glyphosate as a sole source of nitrogen or phosphorous, however, in natural ecosystems, their efficiency in glyphosate degradation may drop significantly because, in the majority of cases, the expression of some enzymatic complex (Figure 1) is activated only in response to a specific intracellular deficit and a specific deficiency of nitrogenous or phosphorous, all conditions mentioned could not be typically found in glyphosate polluted environments. Remotion of glyphosate from water polluted by biological treatment is summarized in Table 1.

### 2.2. Psychochemical Treatment Processes

Physicochemical processes are efficient and economical methods in pollutant removal from water and wastewater. Various physicochemical processes such as adsorption, membrane filtration, and coagulation have been studied at laboratory and pilot scales with the common target of removing glyphosate from water. However, adsorption and membrane filtration technologies have been widely studied. Some studies about the remotion of glyphosate from water polluted by psychochemical processes are summarized in Table 2 and Table 3.

#### 2.2.1. Adsorption

Adsorption has been widely studied due to its simplicity, low-cost, easy operation with high processing efficiency and high removal rates of most pollutants [16]. The key factor in this process is the adsorbent material selection, its performance is influenced by contaminant concentration, pH, adsorption time, temperature, adsorbate doses and ion strength [16]. Over optimal conditions, the glyphosate adsorption mechanism occurs by means of physical and chemical interactions between functional groups in the glyphosate molecule (-COOH, -NH_2_, and -PO(OH)_2_) and adsorbent surface.

- Physical adsorption is mainly caused by the forces of molecular interactions including permanent dipole/induced dipole, Van der Waals dispersion forces as well as hydrogen-bond with acid groups like -OH and -COOH on the material surface [16], but also it is caused by diffusion through a larger number of micro-, meso- and macro-pores with high pore volume [49].
- Chemical adsorption occurs due to electrostatic attraction between the glyphosate molecule and adsorbent surface, which is strongly influenced by pH conditions. In acidic conditions, glyphosate shows a negative charge, so it is attracted to the material’s surface with a positive charge [16,49]. Additionally, onto iron-based adsorbents, the phosphonyl group of glyphosate molecule can make complexation with Fe^2+^ and Fe^3+^ ions forming stable single-tooth or bidentate complexes, thus facilitating the adsorption significantly [16].

**Figure 2:**
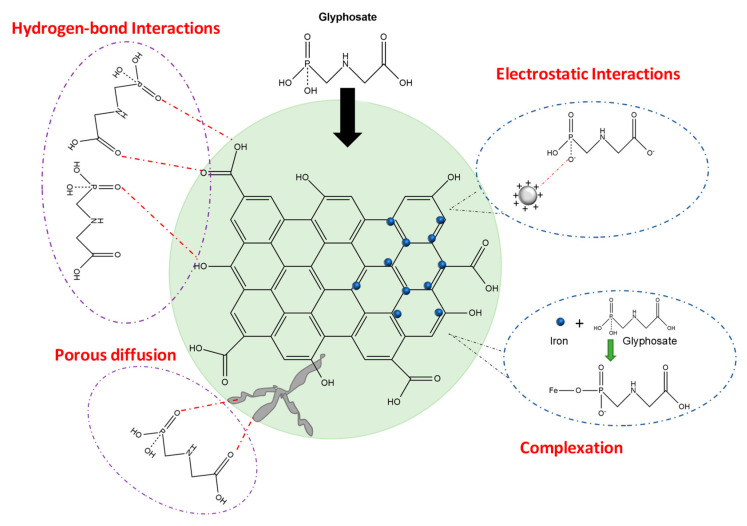
Glyphosate adsorption mechanism of carbon absorbents and iron-based adsorbents.

A graphical representation of possible mechanisms for glyphosate adsorption is illustrated in Figure 2.

Many adsorbent materials have been studied for removing glyphosate from water and wastewater (Table 2). Current adsorption materials include activated carbon [50], bio-carbon [38], zeolite [36], goethite [50], alum sludge [33], graphene oxide and iron-based adsorbing materials [16]. Activated carbon was the first adsorbent studied for glyphosate remotion, it has only reached a range of efficiency of 12–20% [50,51]. However, under optimal parameters, a current study had reached the maximum removal capacity and efficiency of 98.45% using coconut shell activated carbon [35]. Activated bio-carbon produced from rice husk (RHBC) and wood biochar have shown 82% and 100% as maximum removal efficiency at pH 4.0 and pH 5.0 respectively [38,50]. Both adsorbents have shown that adsorption capacity reduces significantly with the increase in pH. On the other hand, glyphosate adsorption using both zeolite (Al) and goethite (Fe) mineral surfaces is generally explained by the formation of complexes [52]. Recently, a study has modified zeolite with cupric ion (Cu-zeolite 4A) in order to improve adsorption capacity; the maximum adsorption capacity for Cu-zeolite 4A was 112.7 mg·g^–1^ based on the Langmuir model [36].

Alum sludge has exhibited the potential to be an efficient and cost-effective adsorbent for glyphosate removal with excellent immobilization ability, two studies have used it as an adsorbent. The first study reported a maximum glyphosate adsorption capacity calculated from Langmuir’s isotherm of 85.9 mg·g^−1^ for dewatered alum sludge (DAS) and 113.6 mg·g^−1^ for liquid alum sludge (LAS), with a removal efficiency of 91.6% and 97.4% respectively [33], and the second exhibited an average glyphosate removal of 99.8% over 10-week test period [35]. Additionally, graphene oxide has excellent adsorption performances for many pollutants, but it is difficult to be recovered from the water after the treatment process. Thus, it is combined with magnetic iron-based adsorbents for an easy separation process. For instance, nanoscale graphene oxide combined with Fe_3_O_4_ to obtain magnetic reduced graphene (RGO/Fe_3_O_4_); could achieve a satisfactory adsorption performance for low GLY concentrations (1–40 mg·L^−1^) with removal rates higher than 86% by adding 10 mg of RGO/Fe_3_O_4_ [16], and graphene oxide combined with magnetic nanoparticles of iron oxide to obtain graphene oxide functionalized by magnetic nanoparticles of iron (α-γ-Fe_2_O_3_); which after 2 h of equilibrium time the maximum removal was 92% at 15 °C [37].

Montmorillonite is a 2:1 type aluminosilicate that has been proposed as an adsorbent for glyphosate removal. Glyphosate molecules accommodate between the layers which increases the interlayer space, XRD patterns show a displacement in the peak that corresponds to (001) plane due to the intercalation compound formation. Adsorption of glyphosate in montmorillonite may be enhanced by the presence of Fe(III), the maximum adsorption capacity was greater than 210 mg·g^−1^ showing great potential for high glyphosate concentrations removal in wastewater [41]. Kaolinite is another clay mineral which can act as GLY adsorbent, GLY interacts with kaolinite surface by the creation of a hydrogen bonds network. Humic acids may enhance the adsorption by the formation of a composite kaolinite-humic acid [42].

As can be observed, even though the adsorption process seems to be an effective way to remove glyphosate from water, the majority of reports have been studied at laboratory scales using synthetic water. Additionally, the main disadvantage of water treatment by adsorption is the residue produced and the not-easy reuse of adsorbents. Thus, in order to scale adsorption technology, it requires to promote the efficiency of adsorbents for glyphosate removal in natural waters and the recovery of adsorbents after-treatment process. Modification of the existing low-cost adsorbents is one of the most significant strategies to improve their performance; however, the main challenge with the use of adsorbents is their low selectivity by co-existing compounds in real water samples. Therefore, the design of an adsorbent with enhanced both capacity and selectivity can be considered as one of the most important research objectives for glyphosate remotion from water. Finally, adsorption treatment has some disadvantages the adsorption efficiency of glyphosate has been better at acidic pH levels, this has implications at the treatment plant application because it determines pH adjustment of the influent. 

#### 2.2.2. Membrane Filtration

Over the last few years, membrane filtration technologies have been used in many areas of water and wastewater treatment. This system works as a barrier for matter transport of an influent stream and separates it into two effluent streams: the permeate and the retentate or concentrate [45,53]. The nanofiltration process has been applied to remove dissolved organic matter, color, and pesticides from aqueous media [46,47]. However, there are very few reports above glyphosate removal using nanofiltration technology. A summary of the removal of glyphosate from water polluted by membrane filtration is presented in Table 3.

**Figure 3:**
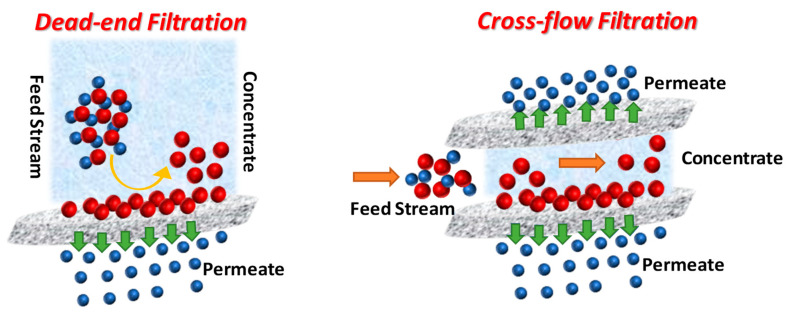
A schematic diagram of glyphosate nanofiltration in aqueous system: dead-end flow and crossflow filtration.

Liu et al. [45] and Saitúa et al. [47] were the first studies about glyphosate removal by nanofiltration process. Liu et al. [45] conducted experiments of glyphosate separation using nanomembrane with cross-flow filtration from simulated wastewater, under experimental conditions (20 °C, pH 2.96, time 30 min, trans-membrane pressure 2.5 MPa) it reached 94.8% of glyphosate retention [45]. On the other hand, Saitúa et al. [47] worked with commercial formulations of glyphosate in synthetic and natural waters using transversal flow nanofiltration treatment plants and reached glyphosate-removal over 80% in distilled water and a similar value for river water [47]. Neither of them did include the effect of coexisting matter such as AMPA, humic acid and calcium salts, which are ubiquitous components of aquatic environments and influence the fate and the behavior of micropollutants as well as its removal by nanofiltration [46]. A schematic diagram of the experimental setup is shown in Figure 3.

Therefore, there is a need for evaluating the effect of natural organic matter on glyphosate removal by nanofiltration. Yuan et al. [46] conducted experiments using two types of commercial thin-film polyamide nanofiltration membranes (NFX, NFY) to remove glyphosate and AMPA from simulated water and included the effect of coexisting matters such as AMPA, humic acid and calcium salts (CaCl_2_ y NaCl). The results demonstrated that with an initial concentration of 50 µg·L^−1^ and 2.5 MPa of trans-membrane pressure (TMP), the rejection percent is 82.8–91.5% for glyphosate and 73.5–86.7% for AMPA. Intermediate concentrations of NaCl and humic acid showed little influence on the glyphosate-retention; on the contrary, CaCl_2_ shown a negative effect during the nanofiltration process. However, rejection performance slightly improved when coexisted humic acid and CaCl_2_ [46]. Finally, this work clearly shows that the composition of the water matrices may influence the efficiency of the nanofiltration process in terms of the micropollutants separation.

The modification of adsorbents structure by means of advanced nanocomposite materials to obtain mixed matrix membranes has been also applied in ultra/nanofiltration systems. Hosseini and Toosi [48], synthesized a nanocomposite of graphene oxide and titanium dioxide (GO/TiO_2_) and mixed it with polysulfone membranes (PSf); GO/TiO_2_/PSf membranes were used for dead-end flow ultrafiltration system of some commercial herbicides, one of them was glyphosate, from aqueous solutions. The results showed that blended membranes acted based on the size molecule exclusion mechanism and GO/TiO_2_ nanoparticles in membrane played a key role in the percentage of adsorption of herbicides on GO/TiO_2_/PSf membrane. Glyphosate rejection was 53% working under the following experimental conditions: dead-end flow ultrafiltration system, glyphosate concentracion (20 ppm), TMP 1 bar and constant temperature (25 °C).

In general, filtration by polymeric membranes seems to be an effective method for the removal of pollutants from water, but there is a lack of knowledge about the operation process because neither of the studies has been done in real water. Therefore, there is still room for investigation researching the feasibility of using the nanofiltration process to remove glyphosate from water. Membrane filtration could present some advantages. One of them is that the filtration process does not destroy pollutant molecules so it does not produce harmful intermediates which could be more toxic than original pollutants, another advantage is that the pollutant could be recovered after saturation of membranes. On the other hand, a disadvantage of membrane filtration could be its specificity to reject specific pollutants, but in real conditions, the composition and characteristics of water could suddenly change affecting the efficiency of the filtration process. Therefore, there is a need for evaluating with more detail the effect of natural organic matter on glyphosate removal by nanofiltration and the effectiveness of this treatment for polluted water with commercial formulations because adjuvants (surfactants) could not be rejected if this process were applied in real conditions of polluted water.

### 2.3. Advanced Oxidation Processes (AOPs)

AOPs have been proposed as alternative methods for water and wastewater treatment, which usually operate at or near ambient temperature and pressure conditions, and they involve the generation of powerful oxidizing agents in sufficient quantity to effective water purification [54]. The AOPs gather numerous techniques based on the in-situ formation of strong oxidants. Among, those radicals, the hydroxyl radical (^•^OH) plays a central role in AOPs due to its high standard potentials (2.8 V *vs.* NHE) in acidic media. It is highly reactive and nonselective that can oxidize and decompose organic matter until its total mineralization CO_2_, H_2_O, and its corresponding inorganic salt [54,55]. In the case of glyphosate molecule, its degradation is attributed to the attack of ^•^OH that lead to cleavage of the C–N and C–P bonds to yield intermediaries such as AMPA and sarcosine or final degradation products such as nitrate, ammonium and phosphate ions, carbon dioxide, and water (Reaction 1). Among AOPs strategies employed to remove glyphosate from water are Fenton and Photo-Fenton process, ozonization (O_3_), photocatalysis, photolysis with hydrogen peroxide and UV light (H_2_O_2_-UV), electrochemical oxidation and photoelectrochemical processes [54,55,56]. The mechanism of some AOPs applied to remove glyphosate from water are explained below and some studies about the degradation of glyphosate by AOPs are summarized in Table 4.

C_3_H_8_NO_5_P + ^•^OH → CO_2_ + H_2_O + NO_3_^−^ + NH_4_^+^ + PO_4_^3−^ (1)

#### 2.3.1. Fenton-Based Treatment Process

Fenton and Photo-Fenton processes have the ability to decompose toxic organic molecules in water and wastewaters by means of strong oxidant species produced in aqueous media. The Fenton process utilizes ferrous (Fe^2+^) ion as a catalyst to decompose hydrogen peroxide (H_2_O_2_) and convert it to ^•^OH radicals [75]. The generally accepted mechanism of the Fenton process is based on Reaction 2.

Fe^2+^ + H_2_O_2_ → Fe^3+^ + ^•^OH + OH^−^ (2)

Based on the classical Fenton treatment process and in order to enhance the degradation efficiency, some hybrid processes based on Fenton´s reaction (Equation (2)) such as photo-Fenton system and electro-Fenton system have been proposed [75]. In the photo-Fenton reaction, ultraviolet or visible light irradiation is applied with the traditional Fenton system with a major purpose of enhancing the production of ^•^OH from photolysis of H_2_O_2_ (Reaction 2) and UV-induced reduction of dissolved Fe^3+^ to Fe^2+^ (Reaction 4) [55].

H_2_O_2_ + *hv* → 2^•^OH (3)

Fe^3+^ + *hv* ⇄ Fe^2^^+^ (4)

In the same context, in electro-Fenton technology, either or both Fenton reagents (H_2_O_2_ and Fe^2^^+^) may be generated through electrochemical reactions, according to Reaction 5 and Reaction 6.

O_2_ + 2H^+^ + 2e^−^ ⇄ H_2_O_2_ (5)

Fe^3+^ + e^−^ ⇄ Fe^2^^+^ (6)

There are a few reports on the above-mentioned Fenton’s reaction applied to remove glyphosate from water, some of them are shown in Table 4. Chen et al. [57], in their study, report a ferrioxalate system under UV irradiation (metal halide lamp, 250 W, λ ≥ 365 nm). Under this system, the degradation of glyphosate was about 60% after 180 min of UV irradiation when the initial concentration of the pollutant was 5.0 mg·L^−1^ and the pH of the solution was 3.5 [57]. Souza et al. [66] studied the degradation of glyphosate by the Photo-Fenton process under optimized conditions at lab-scale (0.27 mmol·L^−1^ of Fe^2+^/Fe^3+^; 1.13 mmol·L^−1^ oxalate; 10.3 mmol·L^−1^ H_2_O_2_ and pH 2.8 ± 0.2, 400 W high-pressure mercury vapor lamp). After 60 min of photo-Fenton process glyphosate concentration fell down below reaching a remotion of 57% of total organic carbon (TOC) and 0.385 mmol·L^−1^ phosphate ion as a final degradation product. Additionally, they reported that the toxicity of effluent decreased from 100% to 54% due to less concentration of glyphosate [66]. Recently, Serra-Clusellas et al. [76] have explored the solar photo-Fenton-like (SPF-like) process for the remotion of 1 mg·L^−1^ of glyphosate and AMPA using low Fe(II) or Fe(III) concentrations. Under these conditions, SPF-like and SPF processes led them to reach 70% and 80% mineralization, respectively.

On the other hand, electro-Fenton-like treatment with Mn^2+^ as a catalyst achieved complete removal of the herbicide (0.1 mM) after 60 min at 200 mA of current applied. Under this process, AMPA was the principal degradation intermediary [67]. Another application of Electro-Fenton was reported by Lan et al. [68] where activated carbon fiber (ACF) was used as a cathode; under optimum operation conditions (t = 360 min; pH = 3; current intensity = 0.36 A; 1 mM of Fe^2+^; pure O_2_ flow rate = 100 mL·min^−1^), it achieved a 50.4% of total organic carbon (TOC) remotion and a 72% of chemical organic demand (COD) remotion. The most recent application of Fenton-process using carbon felt cathode showed that the maximal removal percentage of glyphosate was 91.91% with an applied current density of 10 mA·cm^−2^, pH 3, 0.1 mM Fe^2+^, 0.05 M Na_2_SO_4_, and 0.1 mM as glyphosate concentration under 40 min of treatment. However, only 81.65% of TOC decayed in the same conditions due to the production of some intermediates during the electro-Fenton process that could be more slowly degraded than glyphosate [77]. As has been noted, the Fenton-based process has not been vastly studied as a treatment technology to remove glyphosate from water. This is likely because it requires strict control of operational conditions, as acidic medium (pH 2.8–3.0), and the efficiency of mineralization is not good due to the formation of degradation intermediaries which could be more toxic than glyphosate. Additionally, the Fenton process has been studied only under laboratory conditions using synthetic water, so it requires more studies with water conditions that could be found in real water polluted with glyphosate.

#### 2.3.2. UV-Based Treatment Process

UV-based processes require yields that leads to enough quantities of powerful oxidizing species to degrade pollutants in water. Photolysis (UV/H_2_O_2_; UV/O_3_) and heterogeneous photocatalysis are two UV-based processes studied to remove glyphosate from water polluted [55].

During vacuum-ultraviolet (V-UV, λ = 172 nm) the photolysis of pure water is a particularly interesting or powerful source of ^•^OH radicals that can degrade pollutants in water, according to Reaction 7. According to Azrague et al. [78], this technique presents the advantage of producing ^•^OH without the addition of any supplementary oxidant (e.g., hydrogen peroxide or ozone) or catalyst. However, ultraviolet light combined with hydrogen peroxide (UV/H_2_O_2_) (Reaction 3) or combined with ozone (UV/O_3_) increases the quantity of ^•^OH radicals produced [75]. According to Wang and Xu [55], upon photolysis (λ < 300 nm), O_3_ is decomposed into O_2_ and oxygen atom O(^1^D). O(^1^D) is very energetic and therefore reacts fast with practically all conceivable substrates, including water to form H_2_O_2_ via Reaction 8. To end the reaction process, H_2_O_2_ could form oxidant species based on Reaction 3.

H_2_O + *hv* (V-UV) → ^•^OH + H^•^ (7)

O_3_ + H_2_O + *hv* → H_2_O_2_ + O_2_ (8)

Another treatment process studied to remove glyphosate from water is heterogeneous photocatalysis. The photocatalytic process has been a successful technology for glyphosate degradation, and in this process, the semiconductor catalyst most widely used is titanium dioxide (TiO_2_) [79]. Its photocatalytic activity occurs by photons irradiation with energy equal to or greater at semiconductor *band-gap* energy (hν ≥ Eg), being 3.25 eV for TiO_2_ in anatase phase or 3.05 eV in the rutile phase. The irradiation causes the electrons (e^−^) of the valence band (VB) to exit and migrate to the conduction band (CB) leaving vacancies or positively charged holes (h^+^) in the BV. In this way, electron/hole pairs (e_CB_^−^/h_VB_^+^), where redox reactions are carried out, are generated (Reaction 9). The h_VB_^+^ are strong oxidizing agents where water oxidation reaction form ^•^OH radicals or react directly with pollutants on semiconductor surface (Reaction 10); while the e_CB_^−^ react with electron receptor species such as O_2_ to form superoxide radicals (O_2_^•−^), which also is an oxidant species (Reaction 11) [80].

(9) $${\mathrm{TiO}}_{2}+hv\to {\mathrm{TiO}}_{2({e_{\mathrm{CB}}}^{-}/{h_{\mathrm{VB}}}^{+})}$$

(10) $${\mathrm{TiO}}_{2({h_{\mathrm{VB}}}^{+})}+H_{2}O\to {}^{•}\mathrm{OH}+H^{+}+e^{-}$$

(11) $${\mathrm{TiO}}_{2({e_{\mathrm{CB}}}^{-})}+O_{2}\to {O_{2}}^{•-}$$

Based on the mechanism described above, some studies about glyphosate removal from water by photolysis and heterogeneous photocatalytic process are analyzed below and summarized in Table 4.

Manassero et al. [59] studied the H_2_O_2_/UVC process performed with glyphosate solutions in the concentration of 0.30 mM and 0.45 mM or 50–75 mg·L^−1^, respectively. These glyphosate concentrations were average values of concentration found in wastewaters produced by rinsing herbicide containers [59]. The effects of initial pH, hydrogen peroxide concentration, and incident irradiation were studied. The authors proposed a degradation mechanism where oxidant species are able to cleavage C-P bond to form phosphate as the first step, thus glycine is formed without AMPA and sarcosine generation. Furthermore, formaldehyde, formic acid and nitrate, ammonium, and phosphate ions were detected as final degradation products of glyphosate [59]. Other authors as Vidal et al. [61] and López et al. [62] have studied the H_2_O_2_/UVC system for degradation of commercial glyphosate herbicide mixture in water. Both studies compared the results obtained from a mathematical model with the experimental ones. Vidal et al. reported 80% glyphosate remotion and 70% TOC remotion after 12 treatment hours, these results were similar to the percentage remotion reported by López et al. [62].

About photocatalysis, the study carried out by Assalin et al. [58] showed that heterogeneous photocatalysis using TiO_2_ is effective to remove glyphosate and its degradation intermediaries; after 30 min of photocatalytic treatment of acid glyphosate solution (pH = 10), TOC remotion was 92%. Similarly, Chen and Liu [15] achieved 92% of glyphosate degradation after 3.5 h of irradiation with a starting concentration of 0.25 mmol·L^−1^ and of 6.0 g·L^−1^ of TiO_2_ as an optimum amount of photocatalyst. Additionally, they analyzed the influence of metallic ions and other species (H_2_O_2_, S_2_O_8_^2−^, BrO_3_) in photodegradation efficiency, they have reported that 0.0196 mmol·L^−1^ of Fe^3+^ and 0.01 mmol·L^−1^ of Cu^2+^ were optimal concentrations for the effective remotion of glyphosate from water, whereas optimum values for H_2_O_2_, S_2_O_8_^2−^, and BrO_3_ were 0.1, 1.0, and 0.5 mmol·L^−1^, respectively. Finally, in the study reported by Xue et al. [60], 76% glyphosate degradation was achieved under the following experimental conditions: 0.1 mmol·L^−1^ glyphosate concentration, 1 h of treatment time and synthesized cerium doped TiO_2_ nanotubes by hydrothermal treatment of rutile TiO_2_ nanoparticles followed by posterior impregnation.

Based on UV-based processes analyzed above, only one study reports the formation of degradation intermediates and final products, the rest of them only report the efficiency of glyphosate removal but not the efficiency of mineralization, so in these studies, there could be the formation of intermediate products such as AMPA, sarcosine, glycine, among others. According to Manassero et al, the concentration of wastewater from tank washing is between 50–75 mg·L^−1^; other authors described in this section use a very low concentration of glyphosate. For that reason, it not easy to assume that those processes would be applicable to water treatment on a real scale where glyphosate concentrations could be greater. Furthermore, only one study reports the degradation pathway of glyphosate, showing a lack of knowledge on the degradation route by UV processes. Therefore, more studies are needed with parameters that simulate the real conditions of water in order to study the scaling of UV based treatment processes.

#### 2.3.3. Electrochemical Oxidation Process

Electrochemical Oxidation (EO) is a technology that consists of supplying enough anodic energy to degrade organic matter until its total mineralization or even less complex biodegradable molecules [81]. EO of organic pollutants in water can occur through direct or indirect anodic oxidation reactions. In the first case, pollutants are oxidized by means of direct electron transference from organic pollutants toward the anode surface, which yields very poor decontamination [82]. In the second case, electrochemical oxidation of organic pollutants occurs by electrogenerated species produced by water discharge at the anode surface (M) (Reaction 12), such as physically adsorbed “active oxygen” (physisorbed hydroxyl radical (^•^OH)) or chemisorbed “active oxygen” (oxygen in the lattice of a metal oxide (MO)). The action of these oxidizing species leads to complete or partial decontamination, respectively. Comninellis et al. [81] found that the electrode material nature strongly influences both process selectivity and efficiency; in particular, several anodes favored partial and selective oxidation of pollutants (active anodes), while others favored complete combustion to CO_2_ (non-active anodes).

Active anodes have low oxygen overpotential, in other words, active anodes are good catalysts for oxygen evolution reaction. It is the case of Pt, RuO_2_ or IrO_2_ anodes which interacts strongly with the ^•^OH radicals resulting in the transformation into higher oxide or superoxide chemisorbed at anode surface (MO) (Reaction 13). It allows only partial oxidation of organics (R) forming some organic compounds as short carboxylic acids and others degradation intermediaries, however, it depends on the complexity and stability of pollutants and treatment conditions (RO) (Reaction 14) [82].

M + H_2_O → (M)^•^OH + H^+^ + e^−^ (12)

(M)^•^OH → MO + H^+^ + e^−^ (13)

MO + R → M + RO(14)

In contrast, in non-active anodes, such as PbO_2_, SnO_2_ or boron-doped diamond (BDD), their surface does not provide any catalytic active site for the adsorption of organics from the aqueous medium, so non-active anodes act only as an electron sink for the removal of electrons. In these anodes, the physio-absorbed hydroxyl radicals (M(^•^OH)) remain stable, which allows their availability to achieve complete pollutant’s mineralization (Reaction 15) [82].

(M)^•^OH + R → M + mCO_2_ + nH_2_O+ H^+^ + e^−^ (15)

The process described above does not need to add oxidation catalysts to the solution and does not produce any byproducts. However, indirect electrolysis of pollutants can also occur through the mediation of some electrochemically generated redox reagents. Some of them can be present in effluents or added to the solution as an electrolyte to render the solution conductivity. These redox reagents can act as an intermediary for electrons shuttling between the electrode and the organics. These oxidation mediators can be strong oxidizing chemicals, such as active chlorine, ozone, hydrogen peroxide (H_2_O_2_) and persulfate (S_2_O_8_^2−^), percarbonate (C_2_O_6_^2−^), and perphosphate (P_2_O_8_^4−^) ions; the last ones could be present in the solution according to the following reactions [55,81].

2SO_4_^2−^ → S_2_O_8_^2−^ + 2e^−^ (16)

2PO_4_^3−^ → P_2_O_8_^4−^ + 2e^−^ (17)

2CO_3_^2−^ → C_2_O_6_^2−^ + 2e^−^ (18)

**Figure 4:**
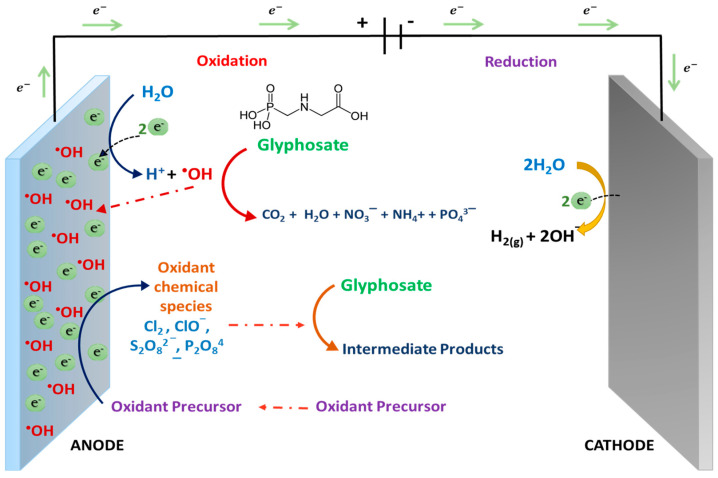
Oxidation mechanism of organic pollutants at non-active anodes.

In the case of chlorine, its natural presence or the deliberate addition of Cl^−^ in a solution can accelerate the process of organic matter degradation. Indeed, the organic matter can be oxidized at the electrode and also in the bulk of solution by chemical reaction with the active chlorine; this reaction is substantially insensitive to electrode surface nature. On the contrary, persulfate, percarbonate and perphosphate (Reaction 16–18) are efficiently generated only using anodes with high oxygen evolution overpotential, such as boron-doped diamond (BDD) or lead dioxide (PbO_2_), especially when oxygen is produced as a secondary reaction. The presence of those strong oxidants in wastewater bulk avoids mass-transfer limitation and increases process efficiency [83,84,85,86]. In summary, Figure 4 presents the electrochemical oxidation mechanism of glyphosate in an aqueous medium.

In spite of the fact that EO has been identified as one of the cleanest technologies to attain glyphosate removal, there are only a few reports about it. Some of them are analyzed below and summarized in Table 4. The study carried out by Neto and De Andrade [69] achieved the complete removal of glyphosate (1000 mg·L^−1^) together with almost total mineralization and a 91% of phosphate production as final degradation product under the following experimental conditions: sodium chloride medium at 30 mA·cm^−2^ during 4 h of electrolysis on RuO_2_ and IrO_2_ dimensionally stable anode (DSA) electrodes [69]. Similarly, Lan et al. [71] studied the electrochemical oxidation process with RuO_2_/TiO_2_ coated titanium mesh as both anode and cathode, assisted with MnO_2_. They reported that glyphosate removal was significantly promoted and most of the released Mn^2+^ ions were oxidatively reverted to MnO_2_, which in turn enhanced the removal of glyphosate, allowing 80% of glyphosate degradation after 120 min of treatment at 10 mA cm^−2^ in 400 mL of solution with sodium sulfate as an electrolyte.

Additionally, Lan et al. [71] have proposed a glyphosate degradation mechanism identifying that glycine and sarcosine were the first intermediaries that were oxidized into oxamic acid and glycolic acid, both of them were finally transformed into acetic acid and NH_3_-N and NO_3_-N. In that way, the authors demonstrated that electro-MnO_2_ is a better process to mineralize glyphosate from water polluted than only the MnO_2_ oxidation process. Other authors reported the combination of adsorption over a nano-metal/resin with advanced oxidation for the degradation of a sample of industrial wastewater polluted with 258 mg·L^−1^ of glyphosate. The maximum degradation rate of glyphosate was enhanced up to 60.5% using only 150 mL of solution in the experimental stage [70]. Recently, the study carried out by Rubí-Juárez et al. [87] proposed a conductive diamond electrochemical oxidation as an alternative to efficiently degrade glyphosate and its organic byproducts. They reported 100% TOC remotion in a solution prepared with pure herbicide, and about 90% TOC remotion in another solution prepared with industrial glyphosate. The electrolysis was carried out in a single compartment electrochemical flow cell with BDD (A = 78 cm^2^) as anode and cathode supplying 100 mA/cm^2^ of current density during 120 min of treatment time, in a solution with 100 mg·dm^−3^ of glyphosate and sodium chlorine as support electrolyte to render the solution conductivity and generated chlorine species. Moreover, the authors reported that the achieved efficiency is due to higher concentrations of hydroxyl radicals and hypochlorite in aqueous media which allowed the formation of PO_4_^3−^, NO_3_^−^ and CO_2_ as the main final products of glyphosate mineralization.

In another hand, the study carried out by Oliveira et al. [88] reported the degradation of real wastewater containing glyphosate using an electrode of PbO_2_ electrodeposited on a three-dimensional matrix of reticulated vitreous carbon (RVC) in a flow reactor. They reported that the optimized values of current density, flow rate, and temperature to lead to an improvement of 70% in the mineralization kinetics were 30 mA·cm^−2^, 3000 mL·min^−1^, and 50 °C, respectively. Additionally, their study reported the importance of mass transfer in anodic oxidation when a low concentration of organics is present in the effluent, so an increase in flow rate could lead to improve the mineralization kinetics, increase the global current efficiency, and decrease the specific energy consumption. Finally, it is important to mention that the study reported by Oliveira et al. [88] is one of the most complete reports because it also presents an economical estimation to treat 1 m^3^ of water, estimating energy consumption of 10–15 kWh·m^−3^ which corresponds to 3.0–4.5 USD·m^−3^.

As can be noticed above, EO could be a promising technology because it lets degrade glyphosate until its total mineralization. Despite that the large-application of EO technology is under development, it has some advantages such as easy operation, environmentally friendly, easy automation, and also could be attractive from an economic point of view because they can use renewable energy from wind and solar sources. However, high cost of electrodes, possibly low conductivity of effluent, electrode fouling, corrosion phenomena, and the possible formation of intermediaries with bigger impact in water ecosystems or human health are some facts that could be considered as disadvantages of process, but also are some challenges to make effort in order to develop a better water treatment technology [73].

#### 2.3.4. Photoelectrocatalytic Treatment Process

Photoelectrocatalysis (PEC) is an emerging technology that combines photocatalysis with anodic oxidation. It is based on the simultaneous application of either a constant anodic potential (E_a_) or constant electric current density (j), and light irradiation (*hv*) to the photoanode [89]. The photoanode is the basis of the mechanism operation of the PEC, this constitutes a material formed by an electrode commonly used in EO on which a semiconductor material with photocatalytic properties is supported. It combination allows that to increase the mineralization capacity of organic pollutants in water [74,88,89]. In this process, the UV or visible light that radiates semiconductor material of photoanode let to occur a photocatalytic process where electron/hole pairs (e_CB_^−^/h_VB_^+^) are generated (Reaction 8, 9 and 10) [90,91,92]. However, in a photocatalytic process, the electron/hole pairs can be recombined; in other words, the e^−^ in the conduction band can return to the h^+^ in the valence band, dissipating energy in form of electromagnetic radiation or heat, it affects the formation of ^•^OH, which are mostly responsible for the degradation of organic pollutants in aqueous medium [91,92,93].

**Figure 5:**
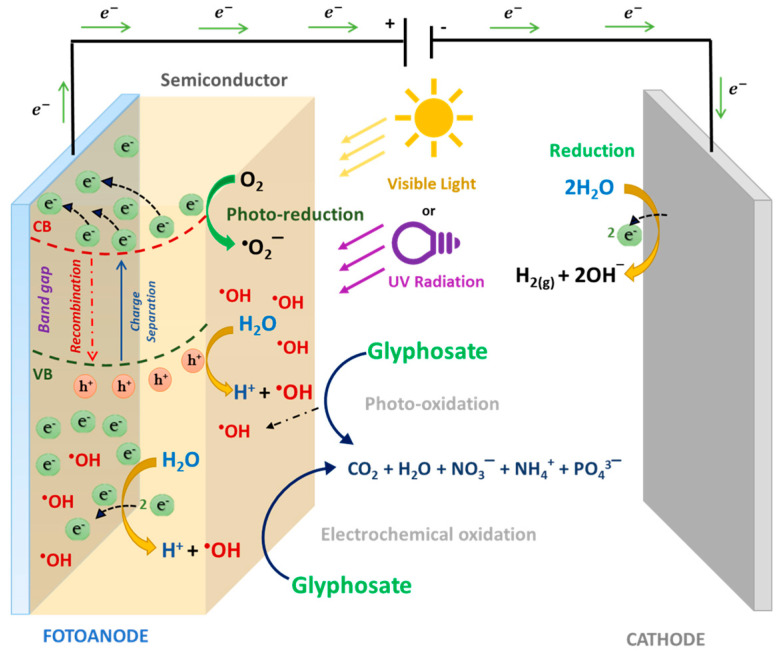
Schematic representation of the mechanism of photoelectrocatalysis applied to organic pollutants degradation.

To avoid recombination and consequently improved the degradation of pollutants in water, the anodic potential or constant current density applied to the photoanode continuously extracts the e^−^ from the BC and sends them to the cathode. In this way, the recombination of e_CB_^−^/h_VB_^+^ pairs is avoided and the generation of a greater amount of h_BV_^+^ in the semiconductor and the subsequent generation of ^•^OH radicals that mineralize organic matter is encouraged. Additionally, because of the anodic potential applied to the photoanode, the electrons from the BV are easily excited and migrate to the BC, causing photocatalytic reactions can occur even with longer wavelengths (visible light instead of UV). Thus, the PEC improves photoanode efficiency and accelerates the degradation of organic compounds compared to photocatalysis and anodic oxidation when they occur independently [90,91,92]. Figure 5 illustrates the mechanism of the PEC process applied to remove glyphosate from water polluted.

Photoelectrocatalysis has been recently proved to remove glyphosate from water; consequently, a few studies have been reported. The first study was carried out by Rubí-Juárez et al. [74], who studied the photoelectrocatalytic process using a conductive boron-doped diamond electrode. The effect of the supporting electrolyte (N_2_CO_3_, Na_2_SO_4_, NaCl) and the applied current density (10–100 mA·cm^−2^) was studied. Results show that photoelectrocatalysis is more efficient to remove glyphosate because of the formation of higher concentrations of oxidant species formed by photoactivation ions electrogenerated by supporting electrolyte. This process reached the complete mineralization of 100 mg·L^−1^ of glyphosate (Round-Up) under the following conditions: current density of 100 mA·cm^−2^, Na_2_SO_4_ as support electrolyte, 140 min of treatment time, and Hg vapor UV light lamp (λ = 254 nm) with an intensity of 930 μW·cm^−2^ [74]. The most recent study is from Sánchez-Montes et al. [73], who also reported that the electrochemical advanced oxidation combined with UVC light irradiation (photo-electro-chemical, P-EC) is an interesting option for water treatment. They assessed the performance of DSA and BDD anodes. The results show that after 1 h of treatment, a complete conversion of the glyphosate herbicide to CO_2_ is only attained with the DSA^®^ electrode and low power UVC sources (9 W). Complete mineralization of glyphosate was also confirmed by the production of NO_3_^−^ and PO_4_^3−^ ions close to the stoichiometric amounts as predicted by the theoretical equation. Additionally, energy consumption as low as 1.25 kWh·g^−1^ was attained [73].

As can be noticed, the few studies about photoelectrocatalysis show that it is an effective combined process to mineralize glyphosate in water. However, the studies reported at the current time do not use a photoanode or combine an electrode with photocatalyst but rather are based on radiate an electrode or solution to promote the formation of oxidant species by means of oxidation of ions in electrolytic support. Thus, there is a vast field of research to study PEC to remove organic pollutants in water specifically because PEC is a promising technology that would operate even under solar light, replacing and reducing treatment cost of operation. Finally, is important to mention that PEC is an emerging technology so there are new studies about PEC reactors and photoanode materials applied to remove other organic pollutants principally dyes or phenolic compounds [81]. All of those studies could be the start point to develop a new system to remove glyphosate from water, this will allow solving environmental problems especially in agricultural areas or zones where aerial fumigations of glyphosate persist.

### 2.4. Combined Treatment Processes

Biological (microorganisms and plants) and physicochemical processes have been combined to treat drinking water and stormwater. Some authors have studied the glyphosate removal efficiency through the adsorption process combined with microorganism degradation in biofilters systems.

Zhang et al. [93] and Yang et al. [94] have studied rain gardens or also known as biofilters or bio-retention areas. They consist of a porous soil filter media in shallows basins planted with various types of vegetation. Zhang et al. have obtained a glyphosate removal efficiency above 90% from stormwater, using a biofilter with *Melaleuca ericifolia* on soil (sand, 96%; silt, 0.8%; and clay 3.2%; organic matter soil 0.4%) [93], meanwhile, Yang et al. used a biofilter with plants such as *Eupatorium perfoliatum*, *Tradescantia ohiensis*, *Veronicastrum virginicum*, *Eragrostis spectabilis*, *Sorghastrum nutan*, *Echinacea purpurea* on soil (sand, topsoil, and compost) obtained a glyphosate removal efficiency of 99% [94]. However, the role of plants within biofilters has not been defined yet, only it has been determined that glyphosate can be accumulated in aquatic macrophyte, if it does not exceed lethal concentrations [95]. Additionally, compact biofilters using organic materials (compost, bagasse) as support medium have been studied for glyphosate removal from water. Bagasse-Based biomixtures as potential substrates for biofilters or have reported that 99% of biodegraded glyphosate is attained after 6 months [25]. Vegetated buffer zones (BZ) between agricultural land and surface waters have proved to be effective filters for sediments and sediment-bound nutrients. The study carried out by Syversen and Bechman [96], achieved only 39% of glyphosate removal efficiency. The low removal efficiency of glyphosate is probably due to the adsorption of glyphosate to the smallest particle size fractions, which have lower trapping efficiency in BZ. The most recent study has studied the removal efficiency using pilot pyrrhotite constructed wetland (Pyrr-CW); in this system, the main removal mechanism is adsorption and microbial activity. In nearly one year, the natural pyrrhotite used as a substrate in a pilot constructed wetland removed 90.3% of herbicide and nutrients (total phosphorous and total nitrogen) from synthetic agriculture runoff [97].

Based on results achieved through combined systems (adsorption and microbial degradation), the authors concluded that the removal efficiencies of glyphosate by biofilters could be related to the physicochemical properties of glyphosate. Thus, through biofilters, sorption is the main removal mechanism of lipophilic compounds (K_ow_ > 4.5), whereas biodegradation is the main removal mechanism (<20%) of hydrophilic compounds. Moreover, in the biofilter system, the feasibility of biodegradation or sorption depends on support material would fulfill and residence time.

Other authors have reported the combination of adsorption with AOPs. Zhang et al. [70] combined adsorption over nano-metal/resin with Fenton oxidation for the degradation of a sample of industrial wastewater polluted with 258 mg·L^−1^ of glyphosate. They found that the maximum degradation rate of glyphosate was enhanced by up to 60% [70]. Xing et al. [98] combined catalytic wet oxidation using modified activated carbon as a catalyst in a co-current up-flow fixed bed reactor through combining AOPs and adsorption. They reported that 100% glyphosate remotion and 93% organic compounds for real wastewater polluted with 200–300 mg·L^−1^ glyphosate. On the other hand, although the combination of AOPs and biological treatment has been reported as a promised method for wastewater treatment, there is no literature reported for glyphosate degradation by this combined treatment. Oller et al. [99] reported that combined AOPs-biotreatment technology has been used for the treatment of wastewater containing pesticides or herbicides, textile wastewater paper mill wastewater, olive mill wastewater, etc., to obtain effective treatment performance. This occurs because AOPs, as a pre-treatment, can convert persistent organic compounds into more biodegradable intermediates, which could subsequently be treated by biological treatment to increase performance and decrease cost. Thus, the combined processes could be an interesting technology for glyphosate treatment and can be further studied. A summary of combined treatments to remove glyphosate from water polluted are presented in Table 5.

## 3. Final Analysis and Conclusions

Glyphosate contamination has emerged as an urgent issue and its negative effects on the environment have attracted considerable attention to develop a variety of treatment processes. The treatment technologies summarized in this review focused on the aqueous medium. Biological treatment can be applied for high amounts of wastewater, and are also low cost and eco-friendly but several operational parameters must be strictly controlled to optimize bioremediation of real water polluted with glyphosate, and in some cases, even a water pretreatment could be required. The adsorption process shows great efficiency at low glyphosate concentration, this process could be a cost-effective solution depending on the elected adsorbate and adsorption competition. However, the main disadvantage of water treatment by adsorption is the residue produced and the not-easy reuse of adsorbents. On the contrary, AOPs are effective to remove glyphosate with a shorter treatment time compared to adsorption and bioremediation. Electrochemical oxidation and photoelectrocatalysis are the cleanest processes and could be used for high glyphosate concentration in wastewater, but some aspects of electrode performance and energy consumption would limit its application. However, these are some challenges to make effort in order to develop better water treatment technology. In the same context, combined processes between AOPs and biological treatment constitute an encouraging technology for glyphosate degradation, but there is limited research. The main purpose of this combination is to increase the biodegradability of the effluent by generating smaller intermediates in the AOPs and the biological treatment would complete the degradation.

The degradation mechanism of glyphosate by different treatment processes analyzed in this review show that biological treatment for glyphosate oxidation generally follows two mechanisms related to the cleavage of C-P and C-N bonds attributed to the action of specialized enzymes in biologic organisms as bacterial and fungi species. The difference between bacterial and fungi pathways has been reported by Zhan et al. [7]. Similarly, glyphosate degradation and mineralization by AOPs are related to the cleavage of C-P and C-N bonds by means of hydroxyl radicals. So, glyphosate molecule is attacked by hydroxyl radicals to yield AMPA, sarcosine or some carboxylic acid to finally transform into phosphates, ammonium, and nitrate ions. However, intermediaries and final products formed during treatment time can differ based on operational conditions as treatment time, pH solution, glyphosate concentration, reactor design, among others.

Considering that the scope of this review is exclusively in an aqueous environment, the most applicable treatment method for glyphosate degradation could be the AOPs. These processes would be the most appropriate treatment method when glyphosate is required to eliminate from an aqueous matrix because AOPs are able to oxidize organic pollutants until its total mineralization. Some studies summarized in this work have reported good degradation efficiencies, it is the case of the work reported by Rubí-Juárez et al. [74], where 100% of glyphosate was mineralized by advanced oxidation in the presence of light using BDD anodes. However, AOPs are known to be an emerging technology in the remediation of soils and air polluted with different organic pollutants; glyphosate degradation in soils polluted has not been studied yet. The work reported by Cheng et al. [100] provides a general overview of AOPs to pesticides, polycyclic aromatic hydrocarbons (PAHs), polychlorinated biphenyls (PCBs) and total petroleum hydrocarbons (TPHs) contaminated soils remediation, and the work reported by Nevárez-Martínez et al. [101] provides the efficiency of anodic oxidation to remove organic pollutants in air. Thus, AOPs could be applied in the remediation of glyphosate in any matrix.

Finally, there has been some research assessing the degradation of this important herbicide through the different treatment processes. However, the majority of this research has been developed at a laboratory scale using controlled conditions and some of them have employed glyphosate analytic standard, not a commercial herbicide. Therefore, it is necessary to study the applicability of this process in the treatment of real wastewater. Additionally, further research is still necessary in order to know more precise mechanisms during the glyphosate oxidation process and the toxicity of intermediates subproducts. Additionally, an analysis of energetic consumption and operation costs at a real scale is still necessary in order to develop a water treatment technology that allows solving environmental problems, especially in agricultural areas or zones where aerial fumigations of glyphosate persist.

## Figures and Tables

**Table 1:** Removal of glyphosate from water polluted by biological treatment.

Microorganism (Bacteria and Fungi)	Experimental Conditions	Glyphosate Concentration (mg·a.i.·L^−1^)	Removal (%)	Ref.
*Pseudomonas* sp.; *Bacillus* sp.	Batch culture	50,000–150,000	-	[19]
*Flavobacterium* sp.	Batch culture isolated from activated sludge	-	25.0	[22]
*Agrobacterium radiobacter*	Batch culture isolated from a bench scale sequencing batch reactor	0.001	99.0	[23]
*Pseudomonas* spp.	Biofilter	10–50	90.0–95.0	[24]
Microorganisms attached to bagasse	Biofilter (biomix)	-	99.0	[25]
Native bacteria from seawater	Batch culture	0.01	48.0	[26]
Activated sludge of wastewater treatment plant	Batch culture	100−1000	-	[27]
*Streptomyces* sp., *Bacillus subtilis* and *Rhizobium leguminosarum*	Batch culture	250	89.7 87.6 86.2	[28]
*Geobacillus caldoxylosilyticus*	Batch culture isolated from central heating system water	169.07	-	[31]
Biofilm	Laboratory aquarium	0.01–0.1	Complete dissipation	[32]
*Aspegillus oryzae* A-F02	Batch culture, isolated from an aeration tank of a pesticide factory	1000	-	[20]
*Fusarium oxysporum*	Platform shaker and Batch bioreactor	50	42.0	[29]
*Trichoderma harzianum*	Batch culture	0.01	69.0	[30]

**Table 2:** Removal of glyphosate from water polluted by adsorption process.

Experimental Conditions			Removal (%)	Ref.
Adsorbent	Operating Conditions	Glyphosate Concentration (mg·a.i.·L^−1^)		
10 mg (RGO/Fe_3_O_4_)	Batch scale, pH solutions: 4; Solid/solution ratio: 1 g·L^−1^	40–40	73.0	[16]
Residual sludge from industrial water	-	50–100 200–500	91.6 97.4	[33]
Metal organic framework/grapheme oxide hybrid nanocomposite (UiO-67/GO)	pH solutions: 4; Treatment time: 3 h	2.560	-	[34]
Alum sludge	Filter: Pot test filled with adsorbents	50	99.8	[35]
Cu-zeolite 4A	Batch scale; Solid/solution ratio: 2 g·L^−1^	50–150	-	[36]
GO-α-γ-Fe_2_O_3_	Batch scale; Solid/solution ratio: 0.5–3.0 g·L^−1^	1–80	92.0	[37]
Coconut shell activated carbon and wood biochar	Batch scale; Solid/solution ratio: 11.4 g·L^−1^ and 12.3 g·L^−1^	0.2–20	98.45 100.0	[38]
Nano-CuFe_2_O_4_ modified	Temperature: 25 °C; Treatment time: 4 h; pH solution: 4	600	98.9	[39]
D151 resin preloaded with Fe^3+^	Temperature: 10–40 °C; Treatment time: 24 h; pH solution: 3.35; NaCl Concentration: 16%	500–1100	-	[40]
Montmorillonite- Fe(III)	Batch scale: Fe(III)-glyphosate 1:1 molar ratio; pH > 5.9; Treatment time: 3 h; Agitation speed: 150 rpm	350.0	98.05	[41]
Kaolinite and Kaolinite-humic acid composite	Batch scale; 10 g of sorbent; Agitation speed: 150 rpm; Treatment time: 6 h; Temperature: 28 °C	40.0	-	[42]
Montmorillonite	Ionic strengths of NaCl 0–0.7; pH solution: 2.0–9.0	0–169.07	-	[43]
Zr-based MOFs (NU-1000, UiO-67)	Batch scale; 3 mg of activated MOFs; Treatment Time: 5 h; mechanical shaker: 180 rpm	1.7	-	[44]

**Table 3:** Removal of glyphosate from water polluted by membrane filtration.

Experimental Conditions			Removal (%)	Ref.
Membrane Filtration	Operating Conditions	Glyphosate Concentration (mg·a.i.·L^−1^)		
Organic GK NF membranes	Cross–flow mode system; Temperature: 20 °C; pH solution: 2.96, TMP: 2.5 MPa	500	94.8	[45]
Polyamide membranes: NFX and NFY	Temperature: 25 °C; TMP: 2.5 MPa	0.05 0.049 **	82.8 73.5	[46]
(TFC) Polyamide membrane	Transversal-flow mode system; pH solution: 8.5; TMP: 4–10 bar	48.0	80.0	[47]
GO/TiO_2_/PSf membranes	Dead-end flow mode system; 25 °C, TMP 1 bar	20.0	53.0	[48]

** AMPA concentration.

**Table 4:** Removal of glyphosate from water polluted by advanced oxidation processes (AOPs).

AOPs	Operating Conditions	Glyphosate Concentration (mg a.i. L^−1^)	Removal (%)	Ref.
UV/Ferrioxalate	V = 80 mL (eight quartz tubes/10 mL); pH = 3.5–6.0; UV-vis Lamp 250 W (λ ≥ 365 nm); t = 180 min	1.0–5.0	-	[57]
UV/TiO_2_	V = 400 mL (cylindrical annular-type reactor); pH from 2.0 to 12.0; UV Lamp = 365 nm; illumination time = 1 h	42.25	9.8–50.2	[15]
Photocatalytic degradation(UV-TiO_2_)	V = 200 mL; high-pressure mercury lamp (125 W, λ > 290 nm); amount of catalyst = 0.1 g·L^−1^ of TiO_2_; t = 30 min.	42.3	99.9	[58]
H_2_O_2_/UV	V_reactor_ = 110 cm^3^; [H_2_O_2_] = 75–200 mg·L^−1^; t = 5 h; 2 UV lamp of 40 W	50.0	70.0	[59]
Photocatalysis Ce-TiO_2_	0.15% Ce-TiO_2_ nanotubes annealed at 400 °C; V = 500 mL; t = 1 h; pH = 7; 125 high-pressure mercury lamps.	22.8	76.0	[60]
UV/H_2_O_2_ experimental and mathematical model	V = 2000 mL (quartz cylindrical reactor, 110 mL, with recirculation); flow rate = 5 × 10^−2^ cm^3^·s^−1^; UV Lamp = 253.7 nm; pH = 5.2; [H_2_O_2_] = 0 to 403 mg·L^−1^; t = 12 h	140.0	80.0 GLY 70.0 TOC	[61]
UV/H_2_O_2_	V = 1000 cm^3^; two low-pressure mercury vapor lamps with one emission wavelength at λ = 253.7 nm; Q = 2 L·s^−1^; t = 8 h	30.0	-	[62]
UV/Goethite	incident light intensity 500–2000 W/m^2^; T = 20 °C, pH 3–9	10.0	92.0 99.3	[63]
Aeroxide TiO_2_ -P25	Volume 250 mL, stirring 600 rpm, UV-A light 60 W/m^2^ wavelength at λ = 365 nm, Time = 240 min	25.0	100	[64]
Photochemical degradation over CuS/Bi_2_WO_6_	Hierarchical CuS/Bi_2_WO_6_ p-n junction photocatalyst; illumination time: 180 min; 44 W light-emitting diode (LED) light irradiation (*λ* > 400 nm)	16.9	85.9	[65]
Photo-Fenton	V = 50 L; closed recirculating system at a flow rate of 2.37 L·min^−1^; [Fe^2+]^ or [Fe^2+^/Fe ^3+^] = 0.27 mmol·L^−1^; [H_2_O_2_] = 10.3 mmol·L^−1^; pH 2.8 ± 0.2	100.0 100.0	- -	[66]
Electro-Fenton Mn^2+^	V = 200 mL; 100 mA constant current; catalyst = 0.1 mM Mn^2+^	22.8	92.0–100.0	[67]
Electro–Fenton	t = 360 min; pH = 3; current intensity = 0.36 A; 1 mM of Fe^2+^; pure O_2_ flow rate = 100 mL·min^−1^	22.8	-	[68]
Electrochemical oxidation with RuO_2_/IrO_2_ electrodes	i = 50 mA·cm^−2^; t = 4 h; electrode composition = Ti/Ir_0.30_Sn_0.70_ O_2_;	1000.0	24.0	[69]
Adsorption and POA’s (H_2_O_2_)	V = 150 mL of glyphosate residue solution; pH = 2–4; adsorbent = nano-tungsten/D201 resin + H_2_O_2_	258.0	60.5	[70]
Electrochemical degradation with MnO_2_	V = 400 mL; acidic pH; i = 10 mA·cm^−2^; t = 120 min	22.8	80.0	[71]
Electrochemical degradation	Anode: Ti/PbO_2_; pH: 3–10; current intensity: 4.77 A; reaction time: 173 min; electrolyte: Na_2_SO_4_	4–16	95.16	[72]
Electrochemical oxidation BDD	Electric charge = 6.0 Ah·dm^−3^; glyphosate pure; t = about 150 min; Chloride media	100.0	- -	[73]
Photochemical Oxidation with BDD	UV lamp (λ = 254 nm); i = 100 mA·cm^−2^; t = about 200 min; supporting electrolyte = NaCl	100.0	-	[74]

**Table 5:** Removal of glyphosate from water polluted by combined treatment methods.

Treatment Technology	Treatment Process Associated	Glyphosate Concentration (mg·a.i.·L^−1^)	Removal (%)	Ref.
Vegetated buffer zones	Adsorption in organic components and clays	0.015–0.030	39	[96]
Biphasic rain garden	Adsorption and microbial degradation	35–1500	99	[94]
Biofilters with plants	Adsorption mixed with microbial degradation	0.0001–0.25	90	[93]
Constructed wetlands	Adsorption and microbial activity	-	90.3	[97]
Adsorption and POA’s (H_2_O_2_)	V = 150 mL of glyphosate residue solution; pH = 2–4; adsorbent = nano-tungsten/D201 resin + H_2_O_2_	258.0	60.5	[70]
Adsorption with AOPs	Catalytic wet oxidation using modified activated carbon as a catalyst in a co-current up flow fixed bed reactor;	200–300 mg·L^−1^	100.0 93.0	[98]

## Abbreviations

^•^OH	Hydroxyl Radical
ACF	Activated Carbon Fiber
AMPA	Aminomethylphosphonic Acid
AOPs	Advanced Oxidation Processes
BDD	Boron-Doped Diamond
BZ	Buffer Zones
C-N	Carbon-nitrogen bond
COD	Chemical Organic Demand
C-P	Carbon-phosphorus bond
DSA	Dimensionally Stable Anode
E_a_	Anodic Potential
e_CB_^−^/h_VB_^+^	Electron/hole pairs
EO	Electrochemical Oxidation
EPSP	5-enolpiruvil-shikimato-3-phosphate-synthetase enzyme
FAO	Food and Agriculture Organization
GLY	Glyphosate
GO/TiO_2_	Graphene Oxide and Titanium Dioxide
IARC	International Agency for Research on Cancer
IBT	Immobilizing Bacterial Technology
K_ow_	Octanol-water partition coefficient
MO	Metal Oxide
NHE	Normal Hydrogen Electrode
PEC	Photoelectrocatalysis
PSf	Polysulfone
TMP	Trans-Membrane Pressure
TOC	Total Organic Carbon
UV	Ultraviolet light
WHO	World Health Organization
